# Neuroleptic malignant syndrome associated with second-generation antipsychotics (SGA): An analysis of reported cases in eudravigilance database, 2017-2020

**DOI:** 10.1192/j.eurpsy.2021.2151

**Published:** 2021-08-13

**Authors:** A. Torres, A. Mignano, I. Viseu, L. Rodrigues, T. Herdeiro, L. Silva, V. Afreixo

**Affiliations:** 1 Nursing, Portuguese Red Cross Northern Health School, Oliveira de Azeméis, Portugal; 2 Department Of Education And Psychology Of University Of Aveiro, CINTESIS - UA – Center for Health Technology and Services Research, Portugal (R&D Unit ref. UIDB/4255/2020), Aveiro, Portugal; 3 Department Of Mathematics, University of Aveiro, Aveiro, Portugal; 4 Department Of Biology, University of Federico II, Napoli, Italy; 5 Department Of Medical Sciences, University of Aveiro, Aveiro, Portugal

**Keywords:** second-generation antipsychotics (SGA), EudraVigilance, neuroleptic malignant syndrome, Psychotic disorders

## Abstract

**Introduction:**

Antipsychotic drugs are the cornerstone of the pharmacological treatment of psychotic disorders; however, even with Second-generation antipsychotics (SGA), adverse effects continue to be extremely accentuated and the treatment effectiveness is compromised by low adherence of the patient.

**Objectives:**

Taking into consideration the importance of adverse effects for psychotic therapeutics, this study aims to analyze the adverse effect of the Neuroleptic Malignant Syndrome (NMS) reported in EudraVigilance Database, associated with 3 widely used SGA, Risperidone, Quetiapine, and Clozapine.

**Methods:**

The EudraVigilance Database was analyzed from 09/01/2017 to 31/10/2020 about NMS, associated with Risperidone, Quetiapine, and Clozapine. NMS is the second most reported adverse effect inside the Nervous System Disorders SOC (System Organ Class). There were just considered NMS as suspected adverse effect.

**Results:**

It was observed a general tendency of reduction of NMS reports from 2017 to 2020 (most of them performed by healthcare professionals). Risperidone presented the highest level of reports during this period (more than 350), followed by Quetiapine and Clozapine. The NMS reports were predominantly referred to the male sex, from 18 to 64 years old. Risperidone presented the lowest number of fatal cases of NMS (1), in contrast with 3 reported with Quetiapine and Clozapine. A significant number of patients with Schizophrenia recovered from NMS.

**Conclusions:**

It is important to do clinical monitoring of the NMS, because it is rare, although it has life-threatening consequences. Pharmacovigilance databases are important tools to evaluate the safety of drugs and it must be more widely and efficiently promoted for healthcare and patients use.

**Disclosure:**

No significant relationships.

